# CULTURAL MOTIVATION TO VOLUNTEER AMONG OLDER JEWISH ADULTS: AN EXPLORATORY STUDY

**DOI:** 10.1093/geroni/igz038.2399

**Published:** 2019-11-08

**Authors:** Eireann O’Dea, and Andrew Wister

**Affiliations:** Simon Fraser University, Vancouver, British Columbia, Canada

## Abstract

The physical, mental, and social benefits for older adults who volunteer are well-documented. Absent from this area of research however, is an understanding of volunteer motivation and experiences among culturally diverse older adults. This study addresses this research gap by exploring the volunteer pathways, motivations, and experiences of Jewish older adults in Vancouver, BC, Canada. The Jewish community is notable for possessing high levels of social capital, indicated by close community ties and the large number of faith and culturally based organizations, including community centres, day schools, seniors’ centres, and family service agencies, which provide many opportunities for older adults to volunteer. Despite this, they remain an understudied population. Semi-structured qualitative interviews were conducted with twenty-one older adult volunteers (age 55+), and two paid volunteer staff in the Jewish community. Theoretical concepts including social capital, generativity, and the life course perspective on aging were used to guide interview questions. Data analysis revealed three themes related to cultural motivation to volunteer: 1.) A desire to support the current and future generations of the Jewish community, 2.) To satisfy the “Jewish ethic” of giving back, and 3.) Experiences of discrimination (anti-Semitism) over the life course. Participants frequently volunteered for organizations that supported the infrastructure of the Jewish community. Findings indicate how cultural experiences and values may influence the decision to volunteer and the types of volunteer roles taken on by older adults. Further, they suggest the ways in which cultural and religious generativity may be expressed through volunteerism, a previously unexplored concept.

